# Diagnosis and Management of Severe Water-Related Skin and Soft Tissue Sepsis: A Summative Review of the Literature

**DOI:** 10.3390/diagnostics13132150

**Published:** 2023-06-23

**Authors:** Shanisa Naidoo, Arnold M. Zwane, Ahmed Paruk, Timothy Craig Hardcastle

**Affiliations:** 1 Department of Surgery, University of KwaZulu-Natal, Durban 4001, South Africa; 2 Trauma and Burn Service, Inkosi Albert Luthuli Central Hospital, Mayville 4058, South Africa; 3 Orthopaedics, Addington Hospital, Durban 4000, South Africa

**Keywords:** soft tissue, necrotizing infection, water borne, vibrio, fungal, aeromonas, debridement, negative pressure wound care

## Abstract

Background: Skin and soft tissue infections (SSTIs) are common presentations in the emergency department. However, this is less common after contact with contaminated saltwater or freshwater. This review presents the diagnosis and management of water-related soft tissue sepsis in this vulnerable and difficult-to-treat subgroup of necrotizing soft tissue sepsis. Methods: A summative literature overview is presented regarding bacterial and fungal SSTI after contact with contaminated water, with practical diagnostic and management aspects. Results: The literature indicates that these wounds and infections remain difficult to treat. An approach using appropriate diagnostic tools with both medical and surgical management strategies is provided. Conclusions: SSTIs due to water contamination of wounds involve unusual organisms with unusual resistance patterns, and require a nuanced and directed diagnostic approach with an adaptation of the usual antibiotic or antifungal selection to achieve a successful cure, along with aggressive debridement and wound care.

## 1. Introduction

An ecological disaster is characterized by the World Health Organization as an unprecedented catastrophic event exacerbated by human activity. The recent floods which devastated KwaZulu-Natal in South Africa during 2022 are an example of such an event [1]. The consequences are compounded in lower- and middle-income countries where an existing burden of disease is coupled with a poorly resourced and fragile health care system. The interaction between environment, pathogen, and host aptly describes the epidemiology and inter-relationship of disease and its consequences. Of particular focus, skin and soft tissue infection (SSTI) following environmental water exposure presents a challenge in terms of surgical management and antimicrobial usage. Accordingly, a guideline-based diagnostic approach which identifies and treats common causative organisms, in addition to a multi-disciplinary team approach, will aid the treating surgeon in providing optimal care.

## 2. Principles of Infectious Disease after Hydrological Disaster

SSTIs frequently occur following flooding, and is associated with major morbidity. The “One Health” concept, which includes the triad of pathogen, environment, and host, can be used to aptly describe the dynamics involved in infection after a hydrological disaster [2]. Typically, inoculation occurs following direct contact with a break in the skin and environmental exposure to fresh, salt, or brackish water. A chain of events ensues which causes disease. Infective agents, each with their individual virulence features, characterize typical invading pathogens. They include viruses, bacteria, fungi, and parasites. This article will selectively focus on fungal and bacterial agents, highlighting the diagnostic approach and treatment options.

A susceptible host is typically of poor socio-economic standing, at extremes of age, had limited access to healthcare, and suffered physical trauma following the ecological disaster [3]. Open wounds in communication with effluent provide a common mechanism for disease transmission. SSTIs can affect any part of the body, although the abdominal wall, perineum, and lower limb are more commonly affected. Successful management hinges on prompt recognition and source control aided by the use of appropriate antimicrobial therapy.

A chief consequence of ecological disasters is damage to infrastructure. Access to life-saving healthcare intervention is limited and underpins the challenges faced by a strained health care system. Major infrastructural installments such as dams and roads are destroyed. Impaired access to critically ill victims, due to entrapment or physical infrastructure and health service collapse poses a challenge to healthcare providers in terms of transport and logistics. Additionally, proper management of wastewater in treatment facilities is pivotal in protecting public health [4]. Unfortunately, ecological disasters—flooding in particular—destroys infrastructure. Consequently, untreated effluent adds to the virulence of multi-drug resistant organisms.

SSTI following an ecological disaster typically results in a polymicrobial systemic infection. The appropriate use of antibiotics is crucial, with misuse having the potential to result in antimicrobial resistance [2,5].

For this summative review, the literature was reviewed via a PubMed and Google Scholar search with access to the EBSCO, Elsevier, Springer, and ProQuest databases. The keywords and MeSH terms that were used included “soft tissue” and “sepsis” and/or “necrotizing infection” and/or “water-related skin sepsis”. The current literature of necrotizing soft tissue infections (NSTI), and in particular the diagnosis and management of water-related NSTI, was reviewed and summarized. This is an underreported aspect of NSTI and makes this review timely in the light of the increased incidences of floods and climatic emergencies across the world.

## 3. Pathophysiology of Infection

There is a chain of events that leads to infections with agents including bacteria, viruses, fungi, parasites, arthropods, and prions [6].

Traditionally, NSTIs have been classified into four groups: Type one being the classic polymicrobial type with facultative aerobes and anaerobes; type two being the monomicrobial beta-hemolytic streptococcus type (occasionally with *Staphylococcus aureus*); type three being when water-related bacterial organisms are involved; and type four being when fungal infection is the cause of the NSTI [7]. This discussion will be restricted to bacteria and fungi, focusing on water-related sepsis, thus focusing on NSTI types three and four.

The infective process begins when one of these agents successfully enters the body of the host and multiplies. The entrance to the host and the host pathogen interface usually occurs through mucosa or compromised skin, such as in the case of open wounds. Entry does not always result in infection, but it may just lead to colonization. The difference between infection and colonization is often a matter of circumstance governed by host factors (age, diabetes mellitus, immunodeficiency), agent factors (virulence), and the external environment.

Injuries associated with water exposure include lacerations following cuts by objects that can be found in the water, puncture wounds secondary to items such as fish hooks, bites from aquatic animals, and patients with pre-existing wounds being exposed to water with contaminants.

Individuals that are at risk include people who spend a lot of time in these waters, including swimmers, fishermen, boaters, flood victims (as evident with the two presented cases), rescue workers following floods, people undergoing leech therapy, and patients who are immunocompromised, such as by HIV, diabetes mellitus, or chronic liver disease.

When considering skin and soft tissue sepsis, it is pivotal to understand that human skin serves as the first line of defense against pathogens, and when it is compromised, infecting pathogens may cause tissue necrosis/damage and incite an inflammatory response [5,8]. Some of these pathogens are capable of producing virulence proteins and toxins that are more potent and responsible for the process, or sequela. There are two main classes of toxins, namely endotoxins and exotoxins.

Endotoxins are lipopolysaccharide chains found mainly in gram-negative bacterial cell walls, which may be beneficial by activating the immune system and enhancing T lymphocyte activities [9]. Exotoxins are actively secreted proteins that cause tissue damage or dysfunction [5,9]. These bind to conserved portions of the T cell receptors, and are able to activate a large number of T lymphocytes. This results in a massive release of cytokines, leading to an exaggerated overstimulation of the host’s inflammatory response.

Part of the inflammatory process involves the host response to tissue invasion and damage, the aim of which is to remove the offending pathogen and begin tissue repair. In some circumstances, this response continues unrestrained, and may be a cause of ongoing tissue damage. The tissue becomes devitalized and hypoxia ensues, which predisposes the host to anaerobic infections.

## 4. Site and Classification of SSTIs

SSTIs result from compromised skin that leads to pathogen invasion of skin, subcutaneous tissue, fascia, and muscles. The World Society of Emergency Surgery classifies SSTIs into three main groups: [10].

- Surgical site infections (SSIs), which are further classified into incisional and organ/organ space infections (not truly SSTIs)
- Non-necrotizing SSTIs
- Necrotizing SSTIs (NSSTIs)

Invasion is further categorized into superficial (skin and subcutaneous tissue) and deep (fascia and muscle). Non-necrotizing SSTIs include erysipelas, impetigo, folliculitis, simple abscesses, and complex abscesses. Necrotizing SSTIs include necrotizing cellulitis, necrotizing fasciitis, Fournier’s gangrene, and necrotizing myositis, depending on the anatomic depth and necrosis sites [10,11].

Soft tissue infections following water exposure are relatively uncommon, and are associated with high infection rates and morbidity or mortality compared to non-water-exposed infections [5,12,13]. The broad and infrequent array of microorganisms following water exposure are said to be the drive behind the complexity of these infections.

The first case reports (individual cases and small case series) on trauma associated with water-exposed infections were published in the 1980’s and 1990’s [13]. The 2004 Indian Ocean tsunami and the 2005 Hurricane Katrina disaster demonstrated the potential impact of water-exposed infections on a much larger scale. The floods in Sub-Saharan Africa have also proven to have similar impacts [14].

**Figure 1:**
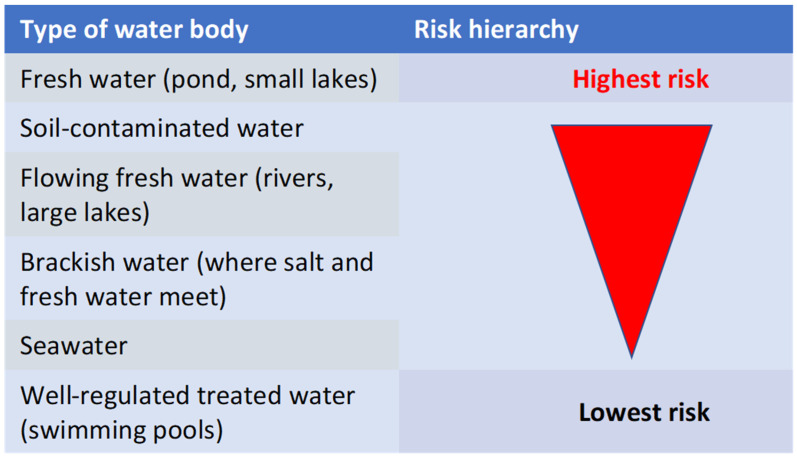
SSTI risk stratification based on exposure of person or wound to different water types.

Different water exposure types hold different infection risks, simply because of the different microorganisms found in different bodies of water [12,13,15]. Bodies of water can be classified into six main groups: one—fresh water (ponds, small lakes), two—flowing fresh water (rivers, large lakes), three—brackish water (where salt and fresh water meet), four—soil-contaminated water, five—seawater, and six—well-regulated treated water (swimming pools). One can appreciate the higher infection risk in fresh waters. Figure 1 illustrates the increasing infection risk depending on water-type.

## 5. Common Microorganisms Linked to Water-Exposed Infections Found in Different Water Bodies

The focus of this discussion is SSTIs in water-exposed wounds. However, it is important to bear in mind the role of colonization and infection by gram-positive *Staphylococcal* and *Streptococcal* organisms, and *Pseudomonas* spp., apart from the organisms that will be discussed below. This also helps to add valuable insight into the management of these patients.

### 5.1. Aeromonas Species (Fresh and Brackish Water)

These are gram-negative, facultatively anaerobic fermentative rods that morphologically resemble the *Enterobacteriaciae* family [9]. *Aeromonas hydrophilia* is the most common species that causes water-exposure associated SSTIs [13,15]. They usually grow at temperatures ranging from 0 to 42 degrees Celsius [9,13]. These are highly virulent pathogens that cause wound infection to develop within 8–24 h of exposure. The infection progresses rapidly to involve fascia, bone, muscle, and joints. *Aeromonas* spp. are inherently resistant to early generation penicillin and cephalosporins, and are preferentially susceptible to ciprofloxacin.

### 5.2. Edwardsiella tarda (Fresh Water)

These are part of the *Enterobacteriaciae* family, and are facultative anaerobic gram-negative rods that usually occur in patients with liver disease or iron overload conditions [15]. They are usually sensitive to most antimicrobials that act against gram negative bacteria.

### 5.3. Vibrio vulnificus (Salt Water, Brackish Water)

These are gram negative, motile, and curved bacteria that are usually associated with the consumption of raw oysters [12]. Infection usually presents with hemorrhagic bullous skin lesions with underlying erythema that can rapidly progress to necrotic ulcers along with a severe septic presentation [12,13,16]. Signs usually develop 12 h after wound exposure, and 25% of these tend to develop into necrotizing infections, osteomyelitis, and/or bacteremia, but may also cause gastrointestinal upset and NSTI in cirrhotics. More than 50% mortality is observed in this scenario [13,16].

### 5.4. Mycobacterium marinum (Salt and Fresh Water)

An atypical mycobacterium that commonly affects fish and rarely humans [12]. This usually presents weeks after wound exposure, and presents as granulomatous ulcerated papules. No systemic disease is usually associated with these bacteria. Surgical intervention is not often required, and these infections are commonly managed with long-term antibiotics.

### 5.5. Eryspelothrix rhusiopathiae (Salt Water)

These are gram-positive bacilli that cause infections, and are usually self-limited in their course [13]. It commonly affects fishermen or seafood handlers, and usually resolves with antibiotic use, but it must be noted that this organism is vancomycin resistant. Usually, no surgical intervention is needed.

### 5.6. Clostridium tetani

This is a large, motile, spore-forming bacterial rod. The primary virulence factor is tetanospasmin, which is a heat-labile neurotoxin that blocks neurotransmitter release for inhibitory synapses. The spores are found mostly in soi, and the tetanus disease is more common in places where access to vaccination or the anti-tetanus toxoid (ATT) is poor [9].

Prevention is essential and involves the use of ATT as a prophylaxis in wound management, specifically in the management of trauma-associated wounds. It is always important to review the immunization status of all patients with trauma-related wounds [17].

### 5.7. Aspergillus Species

**Figure 2:**
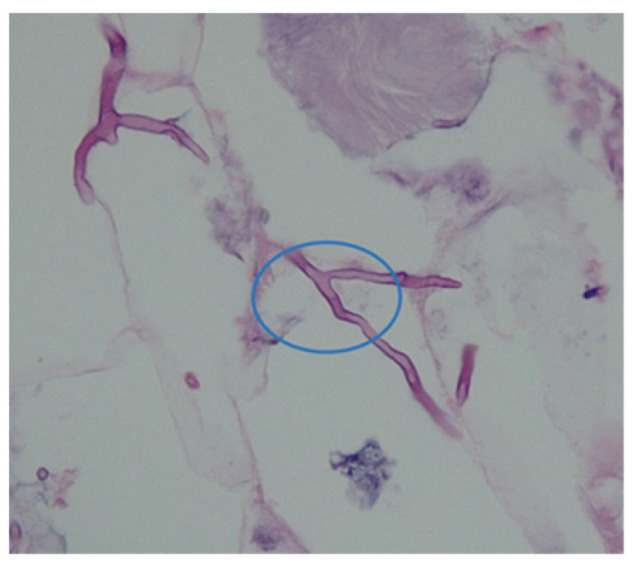
*Aspergillus* septate hyphae found on the wounds of a flood victim treated by the authors.

These are opportunistic mycoses (fungal pathogens). They include multiple species, approximately nineteen in number. They grow as branched, septate, hyphae-producing conidial heads which differ in shape according to the species [9]. This is illustrated in Figure 2.

These pathogens mostly affect patients with immune deficiencies and cause a wide spectrum of disease, including pulmonary infection, abdominal conditions (after ingestion), and superficial cutaneous infections. *Aspergillus* water colonization may involve air conditioning systems, potentially causing nosocomial infections. They can also grow in water distribution systems such as water pipes [18].

**Figure 3:**
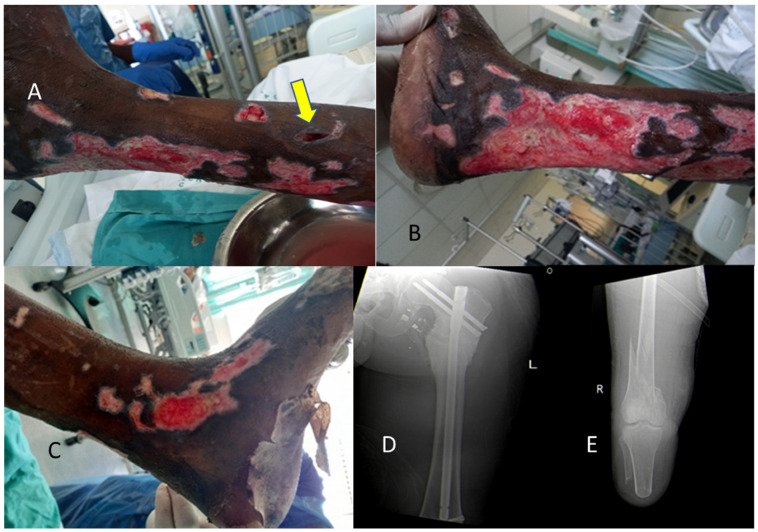
Adult male who had a prior right-side above-knee amputation, and was entrapped in a collapsed structure due to flooding, resulting in crush syndrome and multiple fractures. He presented with multiple septic wounds on day 10 post-injury, and was diagnosed with wound infection by *Aspergillus* fungus, requiring Amphotericin B for six weeks. Images (**A**–**E**)—(**A**) left leg wound with yellow arrow marking abscess site with Aspergillosis; (**B**) leg wound lateral surface; (**C**) medial ankle wound; (**D**) pre-existing left femoral nail after prior gunshot to legs; (**E**) new right femoral fracture of distal third on the side of previous below-knee amputation site (previous gunshot wound with vascular injury). The patient’s wound management included debridement, negative pressure wound therapy, and skin grafting.

Prevention consists of preventing exposure in high risk patients. Decreasing immunosuppression and reconstituting the host’s immune defenses are also important [9]. An example of a recent patient treated by the authors is illustrated in Figure 3.

### 5.8. Rhizopus Species

**Figure 4:**
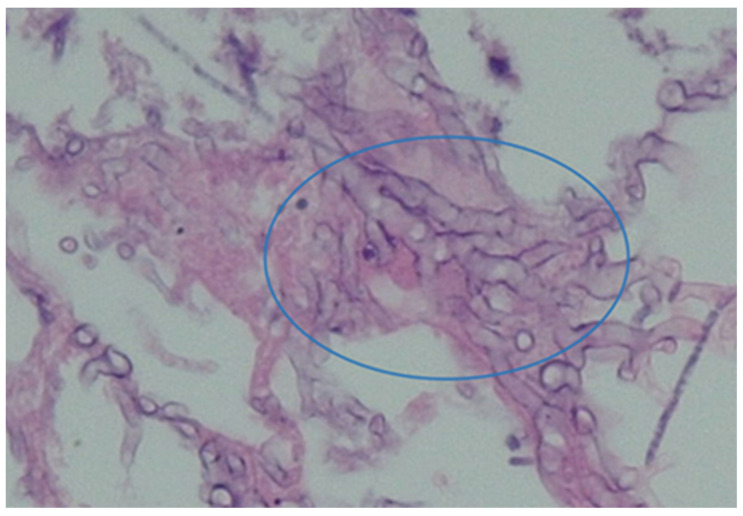
*Rhizopus* species (non-pigmented hyphae) as identified in a recent patient treated by the authors.

These fungi originated from the class of zygomycetes that cause zygomycosis. These appear as ribbon-like, aseptate, nonpigmented hyphae under the microscope (see Figure 4). Infection with these pathogens is rare, however where it occurs, it results in a very high mortality rate, ranging from 70–100% [9].

Infection results in multiple clinical syndromes, which can be pulmonary, angioinvasive, and cutaneous zygomycosis. The source of wound infection is from the contamination of wounds with sporangiospores from the environment, including from fresh water. Cutaneous disease causes nodular lesions with an ecchymotic center. Primary cutaneous zygomycoses occur following traumatic injury, surgical dressings, and burn wound colonization. Early diagnosis is important, involving tissue biopsy for microscopy, histology, and culture (Low sensitivity, and false negatives in up to 50% of mucormycosis cases) [17,19,20,21].

**Figure 5:**
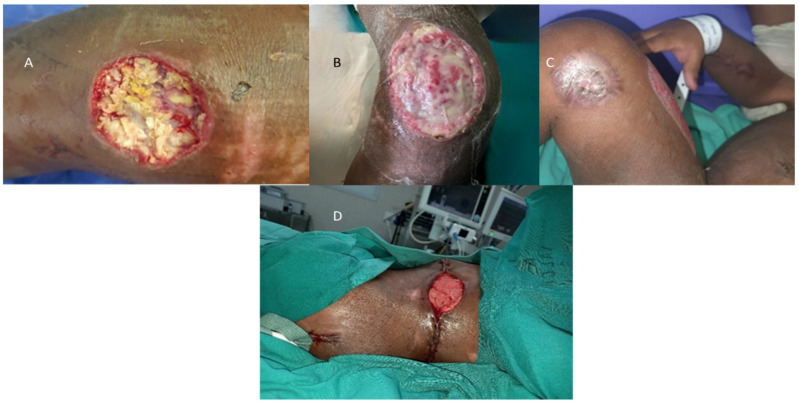
Example of a water-contaminated wound with subsequent fungal infection in a three-year-old after sustaining a brain injury complicated with cortical blindness and a submersion injury, who had left flank and left lower limb degloving injuries and a right femur fracture. He presented with necrotizing fasciitis after being washed away during the recent KZN floods, culturing *Burkholderia epacian*, *Candida parapsilosis*, and *Rhizopus* species. Images (**A**–**D**): (**A**) original knee wound with *Mucormycosis*; (**B**) after debridement; (**C**) healed with skin graft; (**D**) Left flank wound at secondary suture.

An example of such wounds from a recent flood-related admission are demonstrated in Figure 5A–D.

### 5.9. Zoonoses and Vector Borne Diseases

For completion, it is worth mentioning the effect of flood-associated water exposure that leads to zoonosis and vector-borne diseases. Zoonotic disease taeniasis has been frequently associated with flooding. The floodwaters spread disease by transporting taeniasis species’ eggs to areas that were not previously affected. Flooding may also create breeding sites for disease vectors where there are stagnant water pools [14].

There are various other negative health consequences that result from fresh water exposure following flooding, but the focus of this article is SSTIs.

## 6. Diagnosis

Diagnosis is based on three elements, namely clinical examination, imaging, and confirmation of the causative agent (via microbiological culture and sensitivity). However, the diagnosis of NSTIs remains primarily clinical while upholding a high index of suspicion. For clinically uninfected wounds, routine laboratory studies and cultures are not indicated. In clinically *infected* wounds, laboratory studies (full blood count, erythrocyte sedimentation rate, C-reactive protein, and procalcitonin) are reasonable as part of the diagnostic process [17].

Some of these tests also play a significant role in the monitoring of the disease process, i.e., procalcitonin (when available). Blood cultures should be obtained in case of fever or hemodynamic instability, as well as in patients with increased risk of systemic infection. (1-3)-beta-D-glucan indicates an invasive fungal infection; however, the sensitivity and specificity of this marker vary substantially between different fungal populations, and the test is usually unable to detect zygomycetes such as *Mucor* and *Rhizopus* [19,20].

The Galactomannan immunoassay was designed specifically for the easy diagnosis of invasive aspergillosis (IA), but is somewhat insensitive. The assay is the oldest major fungal biomarker test; however, there is relatively little data on its use in children [22,23,24].

Multiple publications exist reporting the utility of nucleic acid-based detection of fungal DNA through PCR, focusing most commonly on *Aspergillus* spp. or *Candida* spp. as pathogens of interest. The T2 Candida assay is an advancement in PCR technology for the diagnosis of candidiasis, although clinical access to this option is limited worldwide. After the DNA amplification step, T2 weighted magnetic resonance is used to detect the amplified products within 3–5 h [22,23,24]. Wound cultures remain the gold standard for establishing the microbiology of the infection and to guide antimicrobial therapy (pus and tissue specimens) [10,11,12,13,15,17].

Different radiological imaging modalities may assist in providing useful information when the diagnosis is uncertain, but these must not delay definitive surgical management [10]. On x-ray, gas can be seen tracking along fascia and subcutaneous planes; however, the absence thereof cannot exclude SSTIs [6,10]. Ultrasounds have the advantage of being rapidly performed at the bedside. Point of care ultrasound (POCUS) in combination with clinical evaluation can improve the diagnosis accuracy for NSTIs [6,10,11]. CTs have higher sensitivity than plain x-rays in identifying NSTIs, and MRI is the imaging modality of choice, though it is limited by emergency availability [10]. The LRINEC score was proposed for predicting the presence of necrotizing infections, with a score of eight or higher having a 75% risk of NSTI. However, recent evidence demonstrates that it lacks sensitivity to be a useful adjunct for the diagnosis of necrotizing infection (Sensitivity of 40.8%) [10].

## 7. Principles of Treatment of Severe SSTIs (NSTIs)

For the management of water-exposed infections, the same basic management principles of skin and soft tissue apply: timely diagnosis identifying patients at risk, appropriate antimicrobials, and timely and appropriate surgical interventions where applicable.

Upon presentation, it must be kept in mind that the majority of patients with water-related SSTIs are trauma patients that have experienced an injury. The approach would then be initial assessment according to the Advanced Trauma Life Support principles and a secondary survey guiding the focused assessment of these wounds/SSTIs [25]. A high index of suspicion is of paramount importance, as all water-exposed wounds are at a higher risk for severe SSTIs [10,12,15].

Early detection of shock and prompt aggressive treatment of the underlying organ dysfunction as per the current Surviving Sepsis guidelines remains an essential component of improving the outcome of critically ill patients [10,26].

**Figure 6:**
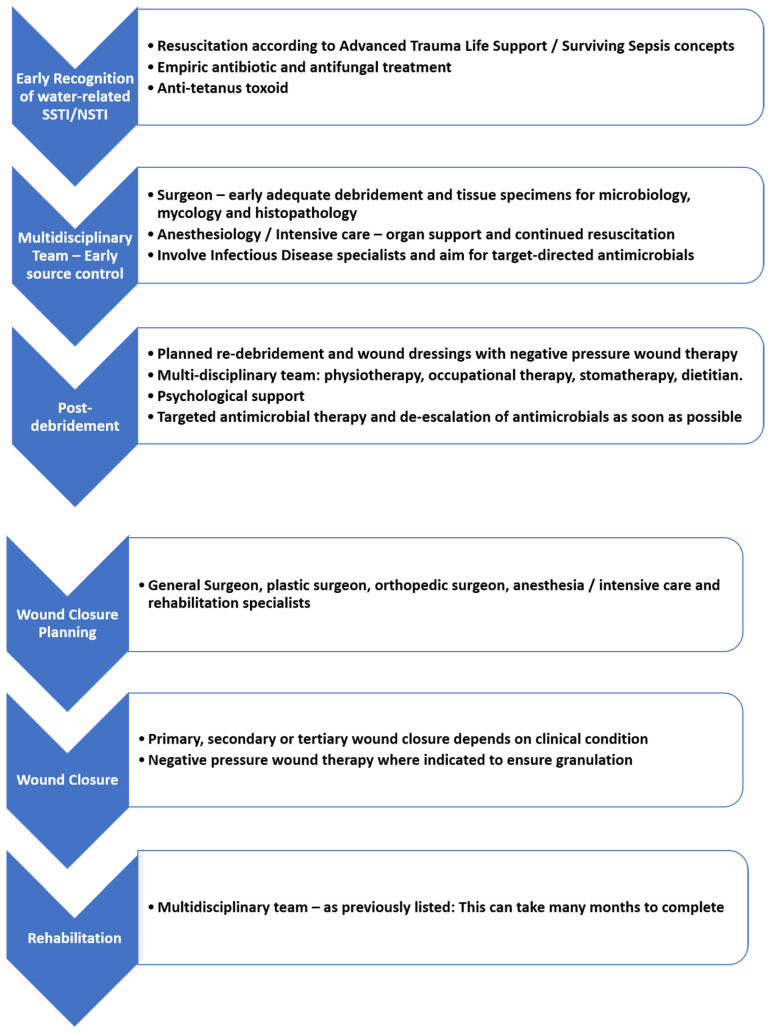
A clinical pathway for the management of NSTI.

Different management strategies have been implemented at different phases. One of the many approaches is provided in Figure 6, which may guide the diagnosis and treatment of such patients.

### 7.1. Acute Phase Management

#### 7.1.1. Resuscitative Measures

Resuscitation should follow the currently accepted “Surviving Sepsis” guidelines and standard trauma or surgical wound management principles [25,26]. Early multidisciplinary care and intensive care admission is recommended.

#### 7.1.2. Prophylactic and Empiric Antimicrobial Therapy

The National Antimicrobial Resistance Strategy Framework is a comprehensive approach which encompasses surveillance, infection prevention and control, and antimicrobial stewardship to combat resistance [27,28]. This is a multidisciplinary approach, and is paramount in the management of any infectious condition, including the topic at hand [27,28].

There is currently no clinical trial data evaluating the benefit of antimicrobial prophylaxis (single dose administration) for patients with open wounds associated with water exposure. Therefore, the use of prophylaxis remains uncertain [17]. Prophylaxis is reasonable in deep wounds beyond the dermis, wounds requiring surgical repair or primary closure, and those associated with crush injuries. Prophylaxis is also advised in wounds with associated vascular or lymphatic compromise, wounds in close proximity to bones or joints, those in special areas (genitals or face), and those in immunocompromised patients. Prophylaxis should be guided by local antibiotic resistance profiles.

Empiric therapy should be based on the epidemiology of the exposure and patient factors, and is usually given for a duration of 3 to 5 days [17]. Regimens are based on the most likely organisms to cause infection [12,13,21]. Initiation is recommended within 1 h, as delays prolong the hospital stay. The lack of active therapy within 48 h of admission is associated with treatment failure [13,29]. Most generic empiric therapy guidelines are tailored for antibacterial cover utilizing appropriate broad-spectrum antimicrobial choice, which covers the type 1 and 2 NSTI gram-positive, gram-negative, and anaerobic organisms [10,11].

For water-exposed wounds presenting as NSTI, the best options for empiric therapy include fluoroquinolones, doxycycline, metronidazole, and clarithromycin in appropriate doses (see Table 1). No guidelines currently include an anti-fungal cover, despite the evidence that wounds exposed to water are colonized with fungi (with or without soil contamination) [12,13,14,15,30,31], which is a shortcoming of the current guidelines. The knowledge of antibiotic pharmacokinetic and pharmacodynamic principles may allow for a more rational determination of optimal dosing regimens in terms of the dose and dosing interval [32,33].

When considering recent experience with flood victims, it is suggested to include an antifungal agent in the empirical management of these severe (polymicrobial) SSTIs. Further research into this aspect of SSTIs is required.

The achievement of appropriate concentrations of the antibiotic at the target site is essential to eradicating the pathogens, and this may be achieved by administration of the first dose via a loading dose. This is essential in the presence of sepsis, as the volume distribution of hydrophilic agents (e.g., Beta-lactams, aminoglycosides) may be altered by changes in the permeability of the microvascular endothelium and alterations in extracellular body water. Loading doses should be appropriate for age and weight, e.g., 2.4 g IVI Amoxicillin Clavulanate in adults. Dose frequency is also related to the concept of tissue-dependent versus concentration-dependent killing. For antimicrobials exhibiting time-dependent activity, the serum concentration should exceed the MIC for an appropriate duration of the dosing interval. Higher frequency dosing, prolonged infusion, and continuous infusions can be utilized to achieve this effect.

#### 7.1.3. Early Surgical Source Control

Source control includes drainage of infected fluid collections, debridement of necrotic, non-viable, septic tissue, removal of infected foreign bodies, amputations, and even correction of any anatomical derangement resulting in ongoing contamination [10,11,13]. Source control is the most important determinant of outcomes in NSTIs. A systematic review and meta-analysis demonstrated that mortality was significantly lower in patients that had surgical intervention within 6 h of presentation, compared to patients delayed more than 6 h. Mandatory re-exploration and repeat debridement should be performed every 12–24 h until the wounds demonstrate little necrosis, or no debridement is required [10,13].

Appropriate, adequate, and efficient debridement saves lives. All devitalized or infarcted skin is removed and healthy, and well-perfused skin is spared. In areas of dubious skin viability, skin preservation and reassessment at the second-look operation is indicated [7,10,11]. All pockets of murky “dishwater” fluid or pus must be explored, deloculated, and irrigated with copious warm saline. The ‘finger test’ may assist intra-operatively to provide tactile evidence of visibly dubious-appearing tissue [9,10]. For perineal wounds, fecal diversion is a useful option. One can make use of closed suction or corrugated drains to allow for draining, irrigation, and easier access to multiple wound tracts and/or cavities. Drains should be anchored on healing skin using simple, non-absorbable sutures. Tissue specimens should always be sent for microbiological culture in normal saline, histopathological investigation in formalin, and for mycology culture in normal saline.

#### 7.1.4. Early Involvement of Critical Care Team

Part of the Surviving Sepsis campaign includes the early admission of patients who are septic or in septic shock, which is recommended within 6 h [26]. Selective patients may benefit from pre-operative resuscitation in a critical care environment prior to source control. This is not always possible, particularly in low-and middle-income countries. In this scenario, regular assessment, evaluation, and appropriate treatment should not be delayed, and should occur independently of patient location. Using dynamic measures to guide resuscitation, thorough physical examination, or static perimeters alone, and also using capillary time as an adjunct is recommended [26]. Critical care aims to optimize organ support by means of mechanical ventilation, cardiac support, and renal replacement therapy if needed.

### 7.2. Post Debridement

During this phase, the wound may need frequent re-exposure and further debridement, thus more short-term dressings may be used. A multidisciplinary approach is of paramount importance in the management of these patients at all stages of wound care [10]. Depending on the timeline, various specialties are involved. Initial treatment always requires coordination between the surgeons, intensivists, and infectious disease specialists. Early rehabilitation is an essential and integral component of recovery, and it would be impossible without the combined efforts by physiotherapy, occupational therapy, dietetics, social workers, and psychological support.

Microbiology results should be available, and directed antimicrobial therapy can be initiated and tailored to microbiology data when available. Severe SSTIs warrant in-hospital management with IV antimicrobials. The switch from IV to oral depends on the clinical response to therapy, their ability to tolerate the switch, and the microbiological etiology. Advantages of the switch are early hospital discharge, reduced isolation of the patient, and an improved quality of life. Patients who are afebrile for over 24 h, have received IV antibiotics for over 24 h, are clinically stable, have no cardiovascular abnormalities, and have a normal white cell count can be changed to oral therapy [28]. Importantly, ongoing organ support may be required from all disciplines concerned.

Table 1 indicates the organisms associated with water-exposed SSTIs and the options for antimicrobials that are likely to cover the organisms [8,12,13,17,18,21].

### 7.3. Intermediate Phase

This phase involves planning for wound closure and involvement of the relevant surgical discipline (e.g., Plastic Surgery) as necessary. In the interim, wound care and support staff play a major role. Wound dressing options are negative pressure wound therapy (NPWT), silver- or alginate-containing dressings, hydrocolloid gels, or paraffin-based dressings. There are many wound care products on the market, so use what is most suited for the specific wound, cost effective, and dressings that the clinician and the team is comfortable with.

The recent addition of instillation and dwell time of the topical wound solution to NPWT is a promising and useful development [34]. NPWT alone has been proven to stimulate blood flow, reduce tissue oedema, and promote granulation tissue formation [9,29,34]. This new addition with the instillation of either normal saline or a topical antiseptic solution is believed to improve the wound bed for optimal healing [34].

Other adjunctive and less conventional treatment options have been explored in an effort to improve outcomes in these patients, including hyperbaric oxygen therapy (HBO) and intravenous immunoglobulin (IVIG) therapy. HBO is a medical treatment that delivers 100% oxygen at a pressure of 2–3 absolute atmospheres, which results in higher tissue oxygen tension. Beneficial effects are noted with improved leucocyte function, inhibition of anaerobic growth, inhibition of toxin production, and enhancement of antibiotic activity; however, there is a lack of high quality or valid research evidence in this regard. HBO could be useful if available, but should not interfere with standard treatment [9,23,35].

IVIG has been postulated to improve outcomes in a selected population of patients, with most studies reporting its use for invasive group A streptococcal infections, including NSTIs, with the proposed action to bind unbound super antigens in super-antigen-mediated toxic shock syndrome. There are currently no guidelines on routine use, and IVIG must be considered on a case by case basis [12,23,35].

### 7.4. Wound Closure Post-Surgical Debridement

Open wounds are managed by making use of three principles: healing by primary, secondary or tertiary intention [34,36]. Primary intention wound closure is usually advocated in clean wounds that have presented for less than 8 h; however, this is not the case in wounds that are water-exposed due to the high risk of infection. Secondary intention encompasses chemical debridement by means of short- and long-term dressings. NPWT has proven to be of great benefit in large, open, and/or exudative wounds. Tertiary intention or delayed closure can be by means of approximating sutures, split-skin grafts, full thickness grafts, flaps, and skin substitutes.

## 8. Conclusions

SSTIs following ecological disasters present a major challenge to healthcare providers and epidemiologists. Medical treatment involves recognition, host optimization, and surgical management. Administration of appropriate antibiotics and strict adherence to antibiotic stewardship principles is crucial. The suggested algorithmic approach may assist in implementation of these pivotal and inter-related aspects in management.

Understanding how components of the epidemiological triad influence each other can aid role-players in developing and implementing interventions to lessen the diseases’ burden. The effects of an ecological disaster are devastating; further research with an emphasis on a multidisciplinary team approach, appropriate medical management, and infrastructural development can reduce morbidity and mortality. Following a diagnostic pathway—and knowledge of the likely organisms—will result in treatment success.

## Figures and Tables

**Table 1:** Common waterborne organisms and likely antimicrobial options.

Pathogen	Antimicrobial
*Aeromonas Species*	Fluoroquinolones: Ciprofloxacin, Levofloxacin 3rd or 4th generation cephalosporins: Ceftazidime/Cefepime
*Edwardsiella Tarda*	Ampicillin Trimethoprim sulfamethoxazole Chloramphenicol
*Vibrio vulnificus*	Doxycycline 3rd or 4th generation cephalosporins Fluoroquinolones
*Mycobactyerium marinum*	Clarithromycin or Trimethoprim Sulfamethoxazole Rifampicin Ethambutol
*Erysipelothrix rhusiopathiae*	Usually self-limiting If required: Penicillin, Cephalexin or ciprofloxacin
*Clostridium tetani*	Prevention with ATT—anti-tetanus toxoid Metronidazole Penicillin Botulinum toxin for tetanus spasms.
*Aspergillus Species*	Amphotericin B Voriconazole for resistant cases
*Rhizopus Species*	Amphotericin B. Posaconazole

## Data Availability

The study only includes new data as images to illustrate the review.

## References

[1] Wikipedia 2022 KwaZulu-Natal Floods. https://en.wikipedia.org/wiki/2022_KwaZulu-Natal_floods.

[2] Iskander K., Molinier L., Hallit S., Sartelli M., Catena F., Coccolini F., Hardcastle T.C., Roques C., Salameh P. (2020). Drivers of Antibiotic Resistance Transmission in Low- and Middle-Income Countries from a “One Health” Perspective—A Review. Antibiotics.

[3] Rose N., Matthäus-Krämer C., Schwarzkopf D., Scherag A., Born S., Reinhart K., Fleischmann-Struzek C. (2021). Association between sepsis incidence and regional socioeconomic deprivation and health care capacity in Germany—An ecological study. BMC Public Health.

[4] Grab S.W., Nash D.J. (2023). A new flood chronology for KwaZulu-Natal (1836–2022): The April 2022 Durban floods in historical context. S. Afr. Geogr. J..

[5] Liang S.Y., Messenger N. (2018). Infectious Diseases After Hydrologic Disasters. Emerg. Med. Clin. N. Am..

[6] Kotra L.P. (2007). Infectious Diseases. xPharm: The Comprehensive Pharmacology Reference.

[7] Bonne S.L., Kadri S.S. (2017). Evaluation and Management of Necrotizing Soft Tissue Infections. Infect. Dis. Clin. N. Am..

[8] Ki V., Rotstein C. (2008). Bacterial skin and soft tissue infections in adults: A review of their epidemiology, pathogenesis, diagnosis, treatment and site of care. Can. J. Infect. Dis. Med. Microbiol..

[9] Murray P.R., Rosenthal K.S., Pfaller M.A. (2008). Medical Microbiology.

[10] Sartelli M., Guirao X., Hardcastle T.C., Kluger Y., Boermeester M.A., Raşa K., Ansaloni L., Coccolini F., Montravers P., Abu-Zidan F.M. (2018). 2018 WSES/SIS-E consensus conference: Recommendations for the management of skin and soft-tissue infections. World J. Emerg. Surg..

[11] Sartelli M., Coccolini F., Kluger Y., Agastra E., Abu-Zidan F.M., Abbas A.E.S., Ansaloni L., Adesunkanmi A.K., Augustin G., Bala M. (2022). WSES/GAIS/WSIS/SIS-E/AAST global clinical pathways for patients with skin and soft tissue infections. World J. Emerg. Surg..

[12] Lewis S., Collins D., Bressler A. (2017). Bacterial Soft Tissue Infections Following Water Exposure. http://www.podiatryinstitute.com/pdfs/Update_2017/Chapter23_final.pdf.

[13] Emigh B., Trust M.D. (2018). Contaminated Wounds: Fresh Water, Salt Water, and Agricultural Contamination. Curr. Trauma. Rep..

[14] Suhr F., Steinert J.I. (2022). Epidemiology of floods in sub-Saharan Africa: A systematic review of health outcomes. BMC Public Health.

[15] Ribeiro N.F.F., Heath C.H., Kierath J., Rea S., Duncan-Smith M., Wood F.M. (2010). Burn wounds infected by contaminated water: Case reports, review of the literature and recommendations for treatment. Burns.

[16] Ruppert J., Panzig B., Guertler L., Hinz P., Schwesinger G., Felix S.B., Friesecke S. (2004). Two cases of severe sepsis due to Vibrio vulnificus wound infection acquired in the Baltic Sea. Eur. J. Clin. Microbiol. Infect. Dis..

[17] Baddour L., Sexton D., Hall K. (2021). UpToDate. www.uptodate.com. https://www.uptodate.com/contents/soft-tissue-infections-following-water-exposure.

[18] Richardson M., Rautemaa-Richardson R. (2019). Exposure to Aspergillus in Home and Healthcare Facilities’ Water Environments: Focus on Biofilms. Microorganisms.

[19] Von Lilienfeld-Toal M., Wagener J., Einsele H., Cornely O.A., Kurzai O. (2019). Invasive Fungal Infection. Dtsch. Arztebl. Int..

[20] Haydour Q., Hage C.A., Carmona-Porquera E.M., Epelbaum O., Evans S.E., Gabe L.M., Knox K.S., Kolls J.K., Wengenack N.L., Prokop L.J. (2019). Diagnosis of Fungal Infections. A Systematic Review and Meta-Analysis Supporting American Thoracic Society Practice Guideline. Ann. Am. Thor. Soc..

[21] Diaz J.H., Lopez F.A. (2015). Skin Soft Tissue and Systemic Bacterial Infections Following Aquatic Injuries and Exposures. Am. J. Med. Sci..

[22] Thompson G.R., Boulware D.R., Bahr N.C., Clancy C.J., Harrison T.S., Kauffman C.A., Le T., Miceli M.H., Mylonakis E., Nguyen M.H. (2022). Noninvasive Testing and Surrogate Markers in Invasive Fungal Diseases. Open Forum. Infect. Dis..

[23] Peetermans M., de Prost N., Eckmann C., Norrby-Teglund A., Skrede S., De Waele J.J. (2020). Necrotizing skin and soft-tissue infections in the intensive care unit. Clin. Microbiol. Infect..

[24] Huppler A.R., Fisher B.T., Lehrnbecher T., Walsh T.J., Steinbach W.J. (2017). Role of Molecular Biomarkers in the Diagnosis of Invasive Fungal Diseases in Children. J. Pediatr. Infect. Dis. Soc..

[25] Committee on Trauma (2019). ATLS Student Manual.

[26] Evans L., Rhodes A., Alhazzani W., Antonelli M., Coopersmith C.M., French C., Machado F.R., Mcintyre L., Ostermann M., Prescott H.C. (2021). Surviving Sepsis Campaign: International guidelines for management of sepsis and septic shock 2021. Crit. Care Med..

[27] (2018). South Africa: South African Antimicrobial Resistance National Strategy Framework [Internet]. www.who.int. https://www.who.int/publications/m/item/south-africa-south-african-antimicrobial-resistance-national-strategy-framework-a-one-health-approach.

[28] Sartelli M., Kluger Y., Ansaloni L., Carlet J., Brink A., Hardcastle T.C., Khanna A., Chicom-Mefire A., Rodríguez-Baño J., Global Alliance for Infections in Surgery Working Group (2017). A global declaration on the appropriate use of antimicrobials across the surgical pathway. Surg. Infect..

[29] Leong H.N., Kurup A., Tan M.Y., Kwa A.L.H., Liau K.H., Wilcox M. (2018). Management of complicated skin and soft tissue infections with a special focus on the role of newer antibiotics. Infect. Drug Resist..

[30] Jenkin A., Mantha P., Palamuthusingam P. (2021). Do we need to change empiric antibiotic use following natural disasters? A reflection on the Townsville flood. ANZ J. Surg..

[31] Baumgardner D.J. (2017). Freshwater Fungal Infections. J. Patient Cent. Res. Rev..

[32] Sartelli M. (2017). Antibiotics Dosing in Critically Ill Patients with Sepsis and Septic Shock [Internet]. Global Alliance for Infections in Surgery. https://infectionsinsurgery.org/antibiotics-dosing-in-critically-ill-patients/.

[33] Urbina T., Razazi K., Ourghanlian C., Woerther P.-L., Chosidow O., Lepeule R., de Prost N. (2021). Antibiotics in Necrotizing Soft Tissue Infections. Antibiotics.

[34] Diehm Y.F., Fischer S., Wirth G.A., Haug V., Orgill D.P., Momeni A., Horch R.E., Lehner B., Kneser U., Hirche C. (2021). Management of Acute and Traumatic Wounds With Negative-Pressure Wound Therapy With Instillation and Dwell Time. Plast. Reconstr. Surg..

[35] Pelletier J., Gottlieb M., Long B., Perkins J.C. (2022). Necrotizing Soft Tissue Infections (NSTI): Pearls and Pitfalls for the Emergency Clinician. J. Emerg. Med..

[36] Chhabra S., Chhabra N., Kaur A., Gupta N. (2016). Wound Healing Concepts in Clinical Practice of OMFS. J. J. Maxillofac. Surg..

